# Pregnancy gestation at delivery and breast milk production: a secondary analysis from the *EMPOWER* trial

**DOI:** 10.1186/s40748-018-0089-x

**Published:** 2018-11-05

**Authors:** Elizabeth V. Asztalos, Alex Kiss, Orlando P. da Silva, Marsha Campbell-Yeo, Shinya Ito, David Knoppert, EMPOWER Study Collaborative Group, Christoph Fusch, Lajos Kovacs, Annie Janvier, Georges Cauotte, Abhay Lodha, Barbara Bulleid, Doug McMilllan, Balpreet Singh

**Affiliations:** 1 Department of Newborn and Developmental Paediatrics, Sunnybrook Health Sciences Centre, University of Toronto, M4-230, 2075 Bayview Ave, Toronto, ON M4N 3M5 Canada; 2 Sunnybrook Research Institute, Sunnybrook Health Sciences Centre, University of Toronto, Toronto, ON Canada; 3 Perinatal and Women’s Health, London Health Sciences Centre, Western University, London, ON Canada; 4 School of Nursing, Departments of Pediatrics, Psychology and Neuroscience, Dalhousie University and Izaak Walton Killam Health Centre, Halifax, NS Canada; 5 Division of Clinical Pharmacology & Toxicology, Department of Paediatrics, Hospital for Sick Children, University of Toronto, Toronto, ON Canada; 6 School of Pharmacy (D.K.), University of Waterloo, Kitchener, ON Canada

**Keywords:** Breast milk production, Domperidone, Mothers of preterm infants

## Abstract

**Background:**

Preterm birth alters the normal sequence of lactogenesis. Lactogenesis I may not yet have started when mothers of very preterm infants (≤ 29 weeks gestation) have given birth. Preterm infants are too small or too ill to initiate suckling in the immediate postpartum period thus altering the normal cascade of event for lactogenesis II. With an increasing demand for mother’s own milk as a primary source of nutritional support in the care of very small and preterm infants, mothers of these infants are often at risk of expressing inadequate amounts of milk. The use of galactogogues is often considered when mothers of preterm infants are still having challenges in breast milk production. What is not clear in the literature is the role that pregnancy gestation at birth plays in successful response to galactogogues. Our objective for this study was to evaluate the role of pregnancy gestation at birth on a mother’s response to the treatment interventions in the ***EMPOWER*** trial.

**Methods:**

For this analysis, the study participants are the 90 mothers who participated in the ***EMPOWER*** trial and were in the stratified in two gestational age groups, 23^0/7^–26^6/7^ weeks and 27^0/7^–29^6/7^ weeks at the time of randomization. The primary outcome measures were the proportion of mothers in each of the gestational age groupings who achieved a 50% increase in breast milk volume on day 14 and day 28 of the study treatment period.

**Results:**

On day 14 of the study treatment, there was no significant difference in the proportion of mothers in the 23–26 weeks gestation group (72.9%) compared to those in the 27–29 weeks gestation group (64.2%), OR 1.51 (95% CI 0.60, 3.78; *p* = 0.38). Similarly, there was no difference in the proportion of mothers between the two gestational age groupings on day 28 of the study treatment, 70.3% compared to 62.3%, OR 1.43 (95% CI 0.58, 3.51; *p* = 0.43).

**Conclusion:**

This secondary analysis was able to demonstrate that mothers of very preterm infants, < 30 weeks gestation at birth, were able to respond to the study treatment in a similar fashion regardless of gestation at birth. If non-pharmacologic approaches are unsuccessful, then a 14–day treatment of domperidone may be considered to enhance breast milk production, even in the lowest gestational ages at delivery.

**Trial registration:**

***EMPOWER*** has been registered at www.clinicaltrials.gov (identifier NCT 01512225) on January 10, 2012.

## Background

Lactogenesis (production and establishment of breast milk) is a complex phenomenon and involves two stages [1, 2]. ***Lactogenesis I*** begins 12 weeks prior to parturition (28 weeks gestation onwards) during which there is an increase in lactose, total proteins and immunoglobulins. The initiation of copious milk secretions is known as ***lactogenesis II*** and occurs within 48–72 h after birth and levels off after day 4 [2]. Preterm birth alters the normal sequence of lactogenesis [3]. Preterm infants are too small or too ill to initiate suckling in the immediate postpartum period thus altering the normal cascade of event for lactogenesis II. With an increasing demand for mother’s own milk as a primary source of nutritional support in the care of very small and preterm infants, mothers of these infants are often at risk of expressing inadequate amounts of milk [4–6]. Although there are several approaches to assist mothers of very preterm mothers to facilitate breast milk production [7], the use of galactogogues is often considered when mothers of preterm infants are still having challenges in breast milk production [8]. What is not clear in the literature is the role that pregnancy gestation at birth plays in successful response to galactogogues.

A recent trial, ***EMPOWER***, sought to determine whether administration of domperidone, initiated within the first 21 days after delivery, would lead to a higher proportion of mothers having a 50% increase in the volume of expressed breast milk at the end of 14 days of treatment (Group A) compared to mothers receiving a placebo (Group B) [9, 10]. More mothers achieved a 50% increase in milk volume after 14 days receiving domperidone (77.8%) compared to those who received a placebo (57.8%); odds ratios [OR] 2.56; 95% confidence interval [CI]1.02, 6.25; *p* = 0.04) [7]. Mothers in this trial all gave birth to their preterm infants before 30 weeks gestation.

For this secondary analysis, we sought to evaluate the role pregnancy gestation at birth played in a mother’s response to the treatment interventions in the trial by comparing the differences seen between the mothers of two gestational age groupings, 23–26 and 27–29 completed weeks.

## Methods

### Trial design

The goal of ***EMPOWER*** was to reaffirms domperidone’s ability to increase breast milk volume to a clinically significant amount which required the use of a modified placebo arm. All enrolled mothers were stratified by gestational age groupings, 23–26 and 27–29 completed weeks gestation at delivery. Mothers were randomly assigned to one of two groups: Group A (domperidone 10 mg orally three times daily for 28 days); or Group B (placebo 10 mg orally three times daily for 14 days followed by domperidone 10 mg orally three times daily for 14 days). The primary outcome for the trial was based on the first 14 days of the study period for both groups.

Mothers were eligible if their preterm infants were born ≤29 completed weeks gestation (23 ^0/7^–29 ^6/7^ weeks); were 8–21 days post-delivery; were pumping a minimum of 6 times a day in the 4 days prior to study entry; and, experiencing a milk volume that was < 150 mL/kg/d (based on their infant’s birthweight) during the previous 72 h period prior to study entry or a maternal report of milk volume reduction by more than 1/3 from a peak volume of the previous 72 h. One year into the study, the inclusion criteria were felt to be too restrictive and were modified to 250 mL/kg/d or experiencing a milk volume reduction of 20% or more from a peak volume during the previous 72 h period prior to study entry.

The trial was conducted in 8 level III Neonatal Intensive Care units across Canada. The research ethics committee of each centre approved the study protocol. All mothers who participated provided a written informed consent before being enrolled. Because the study was utilizing an off-label indication for domperidone, the study was conducted under the Food and Drug Act of Health Canada.

Because of its pragmatic nature, the centres were encouraged to maintain their standard approach in supporting mothers as it related to the types of pumps utilized in each centre. However, for the purpose of the trial, the mothers were encouraged to pump 6–8 times in a given 24-h period. A diary was provided to the mothers for them to record their pumping times, volumes pumped, and any side effects they may experience. Mothers were encouraged to report any adverse events immediately to the study personnel. Non-pharmacologic interventions were encouraged. The trial did not collect serum prolactin.

Because of emerging concerns related to prolonged Q-Tc intervals related to domperidone at the time the study was underway, all mothers had two electrocardiograms performed during the study period: at entry and end of the study intervention period.

### Study participants

For the purpose of this secondary study analysis, the study participants are those mothers who were in the stratified gestational age groups, 23^0/7^–26^6/7^ weeks compared to 27^0/7^–29^6/7^ weeks at the time of randomization.

### Outcome measures

The primary outcome measures were the proportion of mothers in each of the gestational age groupings who achieved a 50% increase in breast milk volume on day 14 of the study treatment period regardless of which treatment arm of the trial they were allocated to.

The secondary outcome measures for each gestational age groupings were: i) the proportion of mothers who achieved a 50% increase in breast milk volume on day 28 of the study treatment period; ii) the mean breast milk volume on days 14 and 28 of the study treatment period; and, iii) the mean percent volume change from the start of the trial to day 14 and from day 15 to day 28 of the study treatment period.

### Analysis

The analysis was carried out using SAS Version 9.3 (SAS Institute, Cary, NC, USA). Descriptive statistics were calculated for all variables of interest. Continuous measures were summarized using means and standard deviations, whereas categorical measures were summarized using counts and percentages. Where applicable, non-parametric statistics (Wilcoxon rank sum test) were utilized.

The primary outcomes were assessed between groups using a logistic regression model. Specifically generalized estimating equations with a logit link function were utilized to account for correlation among observations taken at the same site as well as multiple births by the same mother. Odds ratios were presented along with their associated 95% confidence intervals and *p*-values. Secondary outcomes were assessed between groups using Wilcoxon rank sum tests.

## Results

Between June 1, 2012 and June 30, 2015, 90 mothers were enrolled and equally allocated to the trial study arms in ***EMPOWER***. For this secondary analysis, there were 37 mothers in the 23–26 weeks gestation group (20 in Group A and 17 in Group B) and 53 in the 27–29 weeks gestation group (25 in Group A and 28 in Group B). The mean gestational age were 25.4 weeks and 28.3 weeks respectively. Table 1 outlines the baseline characteristics of the mothers in the two groups. The two groups of mothers were similar in their characteristics except that the mothers in the 23–26 weeks gestation group had a lower rate of being delivered by cesarean section and a lower exposure to antenatal corticosteroids but were not found to be statistically significant. The mean milk volume at the start of the study treatment was similar between the two groups of mothers, 108 ± 96 ml for the mothers in the 23–26 weeks gestation group and 126 ± 95 ml for the 27–29 weeks gestation group.

**Table 1:** Baseline characteristics of mothers

Characteristic	23–26 weeks Gestation	27–29 weeks Gestation	*P*-value
	*N* = 37	*N* = 53	
	n (%)	n (%)	
Maternal age (yr) mean (std)	32.3 (6.6)	30.9 (5.4)	*P* = 0.28
(min, max)	(19.4–44.6)	(19.7–40.9)	
Self-declared ethnicity			*P* = 0.67
Caucasian	23 (62.2%)	36 (67.9%)	
Black	4 (10.8%)	6 (11.3%)	
Asian	7 (18.9%)	9 (16.9%)	
Aboriginal/other	3 (8.1%)	2 (3.8%)	
Smoking prior to pregnancy	10 (27.0%)	18 (34.6%)	*P* = 0.44
Primagravida	12 (32.4%)	19 (35.8%)	*P* = 0.74
Co-Morbidities during pregnancy	*N* = 15 (41.0%)	*N* = 31 (58.0%)	*P* = 0.11
Hypertension (Gestational/chronic)	2	10	
Diabetes (Gestational/Type I and II)	2	6	
Preterm Labour	2	10	
Chorioamnionitis	2	1	
Antepartum haemorrhage	3	7	
Other	8	10	
Antenatal corticosteroids	28 (75.7%)	48 (90.57%)	*P* = 0.08
Cesarean Delivery	16 (43.2%)	32 (60.38%)	*P* = 0.11
Singleton Pregnancy	33 (89.2%)	44 (83.02%)	*P* = 0.55
Milk volume at start of start of trial Mean (std)	108 (96)	126 (95)	*P* = 0.28

Table 2 shows the proportion of mothers in both gestational groups who achieved a 50% increase in expressed milk volume on day 14 of the study treatment. There was no significant difference in the proportion of mothers in the 23–26 weeks gestation group (72.9%) compared to those in the 27–29 weeks gestation group (64.2%), OR 1.51 (95% CI 0.60, 3.78; *p* = 0.38). Because mode of delivery between the groups of mothers was viewed as a potential confounder, a logistic regression was performed after controlling for the mode of delivery with the OR being 1.77 (0.68, 4.58; *p* = 0.24). In addition, there was no significant difference in the proportion of mothers who achieved a 50% increase in milk volume on day 28 of the study treatment in the 23–26 weeks gestation group (70.3%) compared to those in the 27–29 weeks gestation group (62.3%), OR 1.43 (95% CI 0.58, 3.51; *p* = 0.43). There was no difference after controlling for the mode of delivery.

**Table 2:** Primary Outcome

	23–26 weeks Gestation	27–29 weeks Gestation	Odds Ratio (95% Confidence Interval)	*p*-value
	*N* = 37	*N* = 53		
Mothers who achieved a 50% increase in milk volume on day 14, n (%)	27 (72.9%)	34 (64.2%)	1.51 (0.60, 3.78) ^†^	*P* = 0.38^†^
			1.77 (0.68, 4.58)*	*P* = 0.24*
Mothers who achieved 50% increase in milk volume on day 28, n (%)	26 (70.3%)	33 (62.3%)	1.43 (0.58, 3.51) ^†^	*P* = 0.43^†^
			1.38 (0.55, 3.42)*	*P* = 0.49*

^†^*p*-values were based on bivariate logistic regressions

*Adjusted odds ratio and 95% CI, controlling for mode of delivery

Table 3 outlines the mean milk volumes on days 14 and 28 respectively which were similar for both gestational age groupings. Table 4 outlines the mean % volume change from the start of the study (day 0) to day 14 and also from day 15 to day 28 (end of the study treatment). Although the earlier gestational grouping demonstrates a higher per cent volume change, this was not statistically significant.

**Table 3:** Secondary Outcomes: Milk Volumes

	23–26 weeks Gestation	27–29 weeks Gestation	*P* value^a^
	*N* = 37	*N* = 53	
Mean milk volume on day 14 (ml) (mean ± SD)	245.8 (190.4)	242.9 (174.7)	*P* = 0.96
Mean milk volume on day 28 (ml) (mean ± SD)	298.5 (216.0)	285.3 (222.9)	*P* = 0.77

^a^Wilcoxon rank sum test

**Table 4:** Secondary outcomes: volume change

	23–26 weeks Gestation	27–29 weeks Gestation	*P* value
	*N* = 37	*N* = 53	
Mean % volume change day 0 to day 14 (%) (standard deviation)	323.7 (505.8)	149.2 (203.1)	*P* = 0.26
	Median = 103.2	Median = 86.6	
Mean % volume change day 15 to day 28 (%) (standard deviation)	37.9 (62.8)	31.9 (57.1)	*P* = 0.32
	Median = 29.6	Median = 22.9	

## Discussion

Mothers of very preterm infants often have a delay in initiating lactogenesis II. Impaired lactogenesis II is associated with a persistent decreased breast milk volume [11]. Studies have shown that if a mother is producing > 3500 mL/week by the end of week 2 post- delivery, it can be expected that she will produce this ongoing adequate amount into weeks 4 and 5 [12]. For a mother who is producing < 1700 mL/week (an average of 250 mL/day), the outlook is grim with 100% not achieving the goal of 500 mL/day by weeks 4–5 post-delivery. In this study, the mothers in both gestational age groupings had evidence of significant impairment of lactogenesis II with starting breast milk volumes for the trial easily below the 250 mL/day cut-off.

***EMPOWER***, along with previous studies, reaffirmed the effectiveness of domperidone to be considered in mothers who are experiencing challenges with milk production and require a galactogogue [13–16]. This secondary analysis shows that the response is similar between gestational age groupings regardless of whether they were started on a galactogogue earlier versus later in the trial. We were able to show that the mothers in the two gestational age groupings responded similarly to the study treatment in the ***EMPOWER*** study. Although the overall daily volume achieved did not differ between the two groups, there seemed to be a trend of improved volume as demonstrated by the % volume change in the 23–26 weeks gestational age group though this was not statistically significant. However, this may be clinically important as it may suggest that those mothers who may not be physiologically prepared for lactogenesis II may still be able to produce breast milk in clinically adequate volumes for their infants with and without the aid of a galactogogue.

There are numerous maternal and obstetric factors which can modify how lactogenesis may occur in mothers of preterm infants [6]. Two of the more common factors include exposure to antenatal corticosteroids and cesarean delivery. Cesarean delivery is associated with a number of variables which can influence how mothers of preterm infants begin pumping and initiating the cascade of events for lactogenesis [17]. Antenatal corticosteroids have been found to have an effect on reduced breast milk volumes in particular in those women who have delivered at the very preterm gestational age [3]. We did not see an effect on how mothers responded to galactogogue support in this study with either of these factors.

There are strengths and limitations to this study. The findings of this study are based on a population of mothers who were recruited for the purposes of an already published randomized controlled trial [10]. The results of this secondary analysis could be seen as having limited statistical power and be prone to bias. We were reassured that our findings were not overly biased as the two gestational age groups of mothers in the analysis were similar in characteristics and we did not see any effect from any potential confounding variables despite potentially being underpowered.

## Conclusions

This secondary analysis was able to demonstrate that mothers of very preterm infants, < 30 weeks gestation at birth, were able to respond to a galactogogue in a similar fashion regardless of gestation at birth. The use of a galactogogue can be considered if additional support in breast milk production is needed especially in the presence of optimized pumping strategies. Domperidone is, globally, the most widely used galactogogue and, where available, a 14–day treatment of domperidone may be considered to increase breast milk production, even at the lowest gestational ages at the time of delivery. For those countries where restrictions have been placed on domperidone, an alternate to domperidone, metoclopramide, can be considered.

## Abbreviations

CI: Confidence interval
ml: Milliliters
OR: Odds ratio
